# Chemical Sensors for Toxic Chemical Detection

**DOI:** 10.3390/s24186072

**Published:** 2024-09-19

**Authors:** Marijo Buzuk

**Affiliations:** Department of General and Inorganic Chemistry, Faculty of Chemistry and Technology, University of Split, 21000 Split, Croatia; buzuk@ktf-split.hr; Tel.: +385-21-329-474

Industrialization, modern agriculture, urbanization, and modern lifestyles are expected to have a strong impact on the environment. These anthropogenic effects are usually manifested through the ubiquitous presence of toxic and hazardous substances that can pose a serious acute risk to the health of living organisms. In addition, cumulative exposure in trace concentrations can have long-term adverse effects. Accordingly, rapid and accurate detection of harmful and toxic substances, in nature or local environments, can prevent or warn of life-threatening situations or can help to estimate pollutant levels and provide continuous information on concentration fluctuations. To date, numerous reports concerning chemical sensors that provide fast and sufficiently accurate information on the presence of various toxic and harmful substances have been published. The design (architecture) of the chemical sensors depends on various factors, such as the type of target substance, the environment in which the sensors will be used, the purpose of the determination, etc. This leads to different approaches in the development of the sensors, which include the application of different principles, materials, transducers, sensing elements, etc. However, chemical sensors must meet general requirements such as low cost, portability, ease of use, selectivity, sensitivity, durability, etc., which is a constant challenge in this scientific field.

The constant evolution and novel approaches in the development of chemical sensors, together with the large number of analytes, have led to an enormous number of published articles on the detection of toxic chemicals. The Special Issue “Chemical Sensors for Toxic Chemical Detection” is a collection of 11 high-quality original and review articles on current strategies, advances, and challenges in the development of chemical sensors for the detection of toxic and harmful substances.

As a result of the enormous number of articles published, there is a constant need for an up-to-date, comprehensive, and critical insight into the latest trends, findings, and applications of various types of chemical sensors. Accordingly, this Special Issue contains a general review article (contribution 1) focusing on the latest achievements and trends in novel chemical sensor technologies. In this article, the authors focused on recent advances in optical and electrochemical signal-transducing elements in chemical sensors for toxic substances. In addition to “ordinary” biosensors, special attention was given to transcription factor (TF)-based biosensors. Finally, the future prospects for chemical sensors were outlined. The overview presents the latest ideas and advances in this field with 172 references. Due to the great diversity in this field, resulting from the various analytes, design approaches, materials used, detection principles, potential applications, etc., many authors have a specific approach to their review articles. Two specific review articles are published in this Special Issue. Kowalczyk et al. (contribution 2) have summarized the latest reports on the possibility of detecting chemical carcinogens (benzene, formaldehyde, pentachlorophenol, styrene, diazinon) that are involved in the pathogenesis of leukemia by using optical and electrochemical sensors. The 118 up-to-date references guarantee detailed and comprehensive information on the latest trends in the development of optical and electrochemical (bio)sensors for the detection of such chemical carcinogens. Due to the high adsorption energies for NH_3_, large specific surface area, and rich active sites on the surface, two-dimensional (2D) graphene layered structures of transition metal carbons and nitrides (MXenes with the general formula M_n+1_X_n_Tx, where M stands for early transition metal elements, X for carbon or nitrogen, and T for active sites, mainly –Cl, –OH, –O, –F) have been promoted as a selective material for the adsorption of NH_3_. In their review, Cheng et al. (contribution 3) describe, in detail, the synthesis methods and recent advances in the development of gas sensor materials based on Ti_3_C_2_T for NH_3_ detection at room temperature. There is no shortage of critical approach, as the authors discuss the advantages and disadvantages of the various synthesis methods. They also provide an overview of the preparation of composites by adding metal oxides, conductive polymers, and two-dimensional materials to the MXenes, which can improve sensitivity to NH_3_. In addition, the authors summarize and discuss the reports that offer explanations for the detection mechanism. Finally, this review analyzes the challenges and future prospects of Ti_3_C_2_T-based gas-sensitive materials for NH_3_ detection at room temperature.

Furthermore, eight original scientific articles have been published in this issue, three of which present recent advances in sensor technology for gas sensing. Cui et al. (contribution 4) use a series of simulations of the adsorptive system based on WS_2_ to investigate the gas sensitivity to HCHO, CH_4_, CH_3_HO, CH_3_OH, and CH_3_CH_3_. This study revealed a potentially excellent sensitivity and selectivity of WS_2_, which was determined by calculating the density of states (DOS), charge density difference (CDD), work function (W), and current–voltage (I–V) characteristics. Based on the results presented, they concluded that the gas sensitivity of the W defect in WS_2_ can help solve problems such as cross-sensitivity. In addition, they can be useful for integrating the sensor into flexible wearable devices to realize real-time monitoring of individual health and environmental factors. Electrical resistance sensors for the determination of air pollutants (NO_2_, CO, and CH_4_) at room temperature were presented by Sayago et al. (contribution 5). In this research article, the authors used photoactivated ZnO nanoparticles decorated with trivalent ions of rare earth metals as a sensitive material. The effect of photoactivation and the influence of humidity on the analytical signal were investigated. The authors reported that they were able to successfully determine CH_4_ and CO in the ppm range and NO_2_ in the ppb range. The possible mechanisms of detection were discussed with respect to the proposed redox reaction in both dry and humid air. Compared to untreated ZnO, the ZnO decorated with rare earth metals showed significant improvements in analytical performance due to lower resistances and a better response. Finally, the study by Kaya and Ebeoğlu (contribution 6) presents a development of the neural network that responds to gasses such as acetone, ethanol, chloroform, and NO_2_. In this paper, two original studies are presented: the first is the design of an e-nose system consisting of an interdigitated electrode sensor with a bioimpedance spectroscopy (BIS)-based interface circuit to recognize the gasses; the second is the development of a BPNN algorithm to classify the target gas data obtained from the e-nose system with high nonlinearity. The developed neural network model has achieved a test accuracy of 87.16% in classifying NO_2_ (6.7 ppm), acetone (1820 ppm), ethanol (1820 ppm), and chloroform (1465 ppm).

In addition, four reports on the determination of toxic and harmful substances in solutions are presented in this Special Issue. Three articles deal with the development of an electrochemical sensor, while two articles present an optical approach to the detection of toxic and harmful substances. Radić et al. (contribution 7) developed an accurate and sensitive carbon paste electrode, modified with maprotiline tetraphenylborate as a sensing material, to determine maprotiline hydrochloride by potentiometry. This modified carbon paste electrode (mCPE) showed good reproducibility, a fast response, and high accuracy at low concentrations of the analyte, even in a complex matrix. The additional advantage of the proposed sensor is the minimal sample pretreatment during the analytical procedure. An environmentally friendly method for synthesizing cerium tungstate nanoparticles using hydrothermal techniques and their electrocatalytic properties towards hydroquinone were presented by Stanković et al. (contribution 8). The synthesized nanoparticles were also morphologically characterized. By modifying the carbon paste electrode with the obtained cerium tungstate nanoparticles, the authors developed a platform for the voltammetric detection of hydroquinone. Differential pulse voltammetry was proposed as the preferred electrochemical method for the determination, as high linearity and a low detection limit of up to 0.06 µM were achieved. The proposed sensor was used in the development of a method for the detection of hydroquinone in drinking water. The last electrochemical sensor presented in this Special Issue is a highly sensitive method for the detection of Sunset Yellow, based on a graphene-modified glassy carbon electrode (contribution 9). The special feature of this approach lies in the fact that the modification was carried out using a few graphene layers, which were produced by electrochemical exfoliation of graphite rods using current pulses. The electrochemical determination was carried out by cyclic voltammetry, square-wave voltammetry, and amperometry, at various pH values, and in the presence of the interfering species. The developed amperometric sensor shows good linearity over a concentration range between 0.028 and 30 µM, with a limit of detection of 0.0085 µM and a limit of quantification of 0.028 µM. The modified electrode was successfully used to quantify Sunset Yellow in pharmaceutical formulations as well as in chocolate bars and orange juice.

As already mentioned, two optical sensors are presented in this Special Issue. The first article reports on an optical sensor for the detection of glyphosphate pesticides, based on double-emitting carbon dots derived from L-glutathione and formamide, which enables simple and sensitive detection of glyphosate in the sub ppm range in aqueous solutions (contribution 10). The detection method is based on the ratiometric reaction of red to blue fluorescent signals in the presence of glyphosate. Using fluorescence quenching assays, a ratiometric response in the ppm range with detection limits as low as 0.03 ppm is observed. In addition, the authors suggest that other pesticides and contaminants can also be detected in water by appropriately modifying the surface of the carbon dots. Finally, Atchudan et al. (contribution 11) present the hydrothermal preparation of natural nitrogen-doped carbon dots (NN-CDs) synthesized with a green precursor and their application in the detection of Fe^3+^ ions. The authors utilized the fluorescence quenching properties of the prepared material in the presence of Fe^3+^ for their determination. The sensitive and selective sensor platform for Fe^3+^ detection was developed, with the detection limit calculated to be 0.86 µM with a dynamic range of 5–25 µM.

In summary, the publications in this Special Issue provide a comprehensive and critical insight into the recent trends, findings, and applications of different types of chemical sensors for the determination of toxic chemicals. The reviews focus on current topics such as the determination of carcinogenic substances or ammonia. The one review deals with the latest trends and advances in sensor technology. The research articles deal with the determination of harmful and toxic gasses by changing the electrical properties of the sensor materials. The determination of toxic chemicals in water media is presented in three articles, exploring electrochemical methods for the determination of various chemicals, while two reports on the development of novel optical methods are also included.

Finally, I would like to express my personal thanks to all the authors and reviewers who have contributed to this Special Issue. I would also like to thank the staff of the journal Sensors for their continued support and suggestions.

